# Macrophage to neuron communication via extracellular vesicles in neuropathic pain conditions

**DOI:** 10.1016/j.heliyon.2024.e41268

**Published:** 2024-12-17

**Authors:** Francesca Picco, Lynda Zeboudj, Silvia Oggero, Vincenzo Prato, Thomas Burgoyne, Nikita Gamper, Marzia Malcangio

**Affiliations:** a Wolfson Sensory, Pain and Regeneration Centre, King’s College London, London, United Kingdom; b Faculty of Biological Sciences, School of Biomedical Sciences, University of Leeds, Leeds, United Kingdom; c UCL Institute of Ophthalmology, University College London, London, EC1V 9EL, United Kingdom; d Royal Brompton Hospital, Guy's and St Thomas' NHS Foundation Trust, London, United Kingdom

**Keywords:** Neuropathic pain, DRG neurons, Macrophages, Extracellular vesicles, microRNAs, Cytokines

## Abstract

Neuropathic pain following peripheral nerve injury results from maladaptive changes in neurons and immune cells contribution to mechanisms underlying chronic pain. Specifically, in dorsal root ganglia (DRG), sensory neuron cell bodies release extracellular vesicles (EVs) which promote pro-inflammatory macrophage accumulation that facilitates nociceptive signalling. Here, we show that macrophages shuttle EVs to neurons. Indeed, bone marrow-derived macrophages (BMDMs) release EVs containing microRNA-155 (miR-155) which are taken up by cultured sensory neurons. EV-mediated transfer of miR-155 suppresses phosphatase *Ship1* expression and increases cytokine interleukin-6 (IL-6) contents. Intrathecal-injected BMDM-derived EVs accumulate in lumbar DRG and EVs containing miR-155 antagomir result in *Ship1* upregulation, *Il6* downregulation in neurons in concomitance to attenuation of neuropathic mechanical hypersensitivity. These data suggest that, under neuropathic conditions, pro-inflammatory macrophages shuttle EV-containing miR-155 to neurons and contribute to the expression of pronociceptive IL-6 in DRG.

## Highlights

• DRG neurons uptake macrophage-derived EVs containing miR-155.
• MiR-155 suppresses *Ship1* and up-regulates *Il6* resulting in more IL-6 release.
• Intrathecal-injected EVs accumulate in DRG neurons.
• EVs containing miR-155 antagomir attenuate *Il6* expression and neuropathic allodynia.

## Graphical abstract

**Figure:**
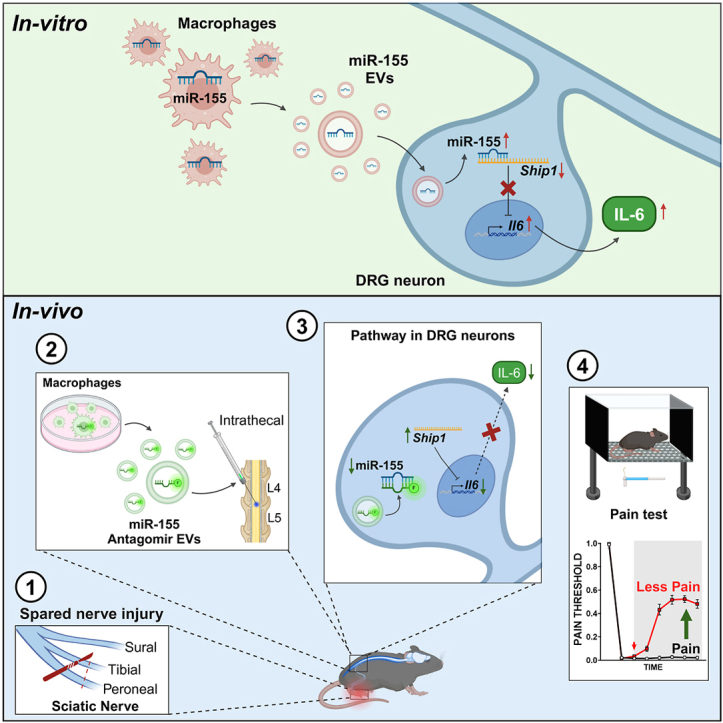

## 1 Introduction

Pain is a universal sensation essential for survival. It is typically acute and serves as a protective mechanism to prevent further injury and promote healing. Pain signalling arises from the detection of harmful stimuli by peripheral terminals of specialized sensory neurons, namely nociceptors, which deliver nociceptive signalling to spinal cord dorsal horn neurons on their way to the brain where pain is felt. Sensory neuron cell bodies reside in the dorsal root ganglia (DRG) and include the nociceptors-small diameter cells with unmyelinated axons that transmit noxious stimuli- and proprioceptors-large diameter cells with myelinated axons that transmit non-noxious stimuli. Neuropathic pain that follows peripheral nerve injury results from changes in both peripheral and central pain pathways and pain often becomes chronic [1]. Existing evidence indicates that immune cells play functional roles in chronic pain mechanisms at several sites along the nociceptive pathways [2]. Specifically, in the DRG, macrophages respond to neuronal cell body activity, accumulate in large numbers and cluster around the cell bodies of injured neurons [3]. Indeed, DRG macrophages, make contacts with sensory neuron cell bodies through pseudopodia, and neurons establish direct communication with macrophages through the exchange of extracellular vesicles (EVs) [4,5]. EVs are lipid-bilayer particles generated by all cells, however loading of cargo depends on cell types and loading of RNA is considered as being a specific and active mechanism although passive loading is also possible [6]. Cargo delivery to recipient cells occurs through direct membrane fusion or endocytic pathways [7] and EV cargoes include small non-coding RNAs (microRNAs, miRs) that repress target mRNA [6]. Relevantly, miR expression is dysregulated in mouse DRG following peripheral axon injury as well as in white blood cells, skin and peripheral nerve of neuropathic patients [8,9]. For instance, in painful neuropathies, sural nerve miR-21 levels are higher than in non-painful neuropathies [10]. In pre-clinical settings, miR-21 is upregulated in injured DRG neuron cell bodies and released in EVs which are engulfed by macrophages where miR-21 promotes M1-like phenotype via inhibition of TGFβ pathway [5]. Such pro-inflammatory macrophages facilitate nociceptive signalling and contribute to neuropathic pain mechanisms [4,5]. Furthermore, in acute inflammatory pain models, there is evidence that anti-inflammatory M2-like macrophages contribute to attenuation of nociceptive processing through transfer of vesicle-containing mitochondria to sensory neurons [11]. Interestingly, miRs regulate macrophage polarisation and inflammatory response with miRs being specifically upregulated or downregulated in M1-and M2-like macrophages [12,13].

Herein, we focus on macrophage communication with sensory neurons in DRG after peripheral nerve injury and show that bone marrow derived macrophages polarised toward M1-like phenotype, release vesicles containing miR-155 that are taken up by sensory neuron in culture. Subsequently, we provide *in-vivo* evidence that delivery of macrophage-derived EVs containing miR-155 antagomir results in accumulation in DRG neuron cell bodies and attenuation of neuropathic allodynia.

## 2 Results

### 2.1 Primary macrophages accumulate extracellular vesicles that contain miR-155

**Fig. 1:**
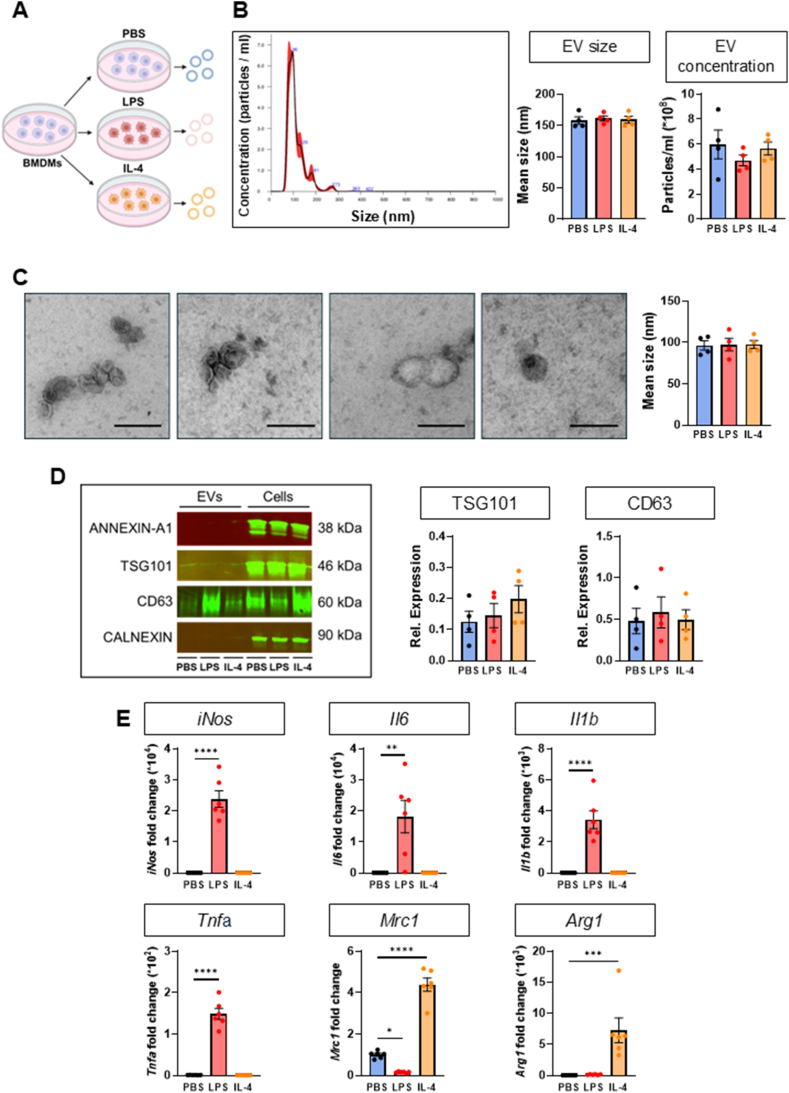
**Primary macrophages release TSG101 and CD63 positive extracellular vesicles.** Data are presented as means ± SEM. ∗p < 0.05, ∗∗p < 0.01, ∗∗∗p < 0.001, ∗∗∗∗p < 0.0001 by One-way ANOVA with Dunnett’s (B,C,E) and Tukey’s (D) multiple-comparison test. **(A)** Schematic representation of cultured BMDMs treatment protocol with vehicle (PBS), LPS (100 ng/ml) or IL-4 (20 ng/ml) for 16 h. **(B)** Representative size distribution histogram (NanoSight^TM^) and quantification of mean size (nm) and concentration (particles\ml) of vesicles obtained at 100,000 *g* for 1 h at 4 °C (n = 4 mice per group). **(C)** Representative transmission electron microscopy images and quantification of mean size (nm) of BMDM-derived vesicles. Scale bar: 200 nm. (n = 4 mice per group). **(D)** Representative Western blots of BMDM-cells and EVs and quantification of ANNEXIN-A1 (38 kDa), TSG101 (46 kDa), CD63 (60 kDa) and CALNEXIN (90 kDa) in EVs plotted as ratio EVs/cells of origin (n = 4 mice per group). **(E)** Quantification by RT-qPCR of polarisation markers fold change over vehicle (PBS)-treated BMDMs compared to treatment with LPS (100 ng/ml) or IL-4 (20 ng/ml) for 16 h (n = 6 mice per group).

First, we confirmed existing evidence that mouse bone marrow-derived macrophages (BMDMs) accumulate extracellular vesicles (EVs) into the culture media [14]. Specifically, overnight treatment of BMDMs with either lipopolysaccharide (LPS, 100 ng/ml) or interleukin-4 (IL-4, 20 ng/ml) resulted in accumulation of EVs in the culture media that displayed an average size of ∼100–150 nm and concentration of about 400 million/ml, which was comparable to control BMDMs (Fig. 1A–C). In further characterisation of BMDM-EVs by Western blot, we observed expression of exosome markers TSG101 and CD63 but neither ectosome marker annexin-A1 nor intracellular marker calnexin, that excludes cell contamination (Fig. 1D).

Then, we confirmed that LPS and IL-4 treatments were associated with intracellular expression of gene markers for M1-and M2-like macrophages. In LPS-treated BMDMs, we observed up-regulation of *iNos*, *Il6* (∼20,000-fold increase), *Il1b* (∼3000-fold increase) and *Tnfa* (∼150-fold) and down-regulation of *Mrc1* but no change in *Arg1*. Instead, in IL-4-polarised cells we found up-regulation of *Mrc1 (*∼4-fold increase) and *Arg1* (∼8000-fold increase) (Fig. 1E).

This data suggests that, independent of polarisation state, BMDMs accumulate small EVs and this enabled us to achieve our main objective to establish whether BMDM-derived EVs are transferred to neurons. We started with an *in-vitro* approach to identify specific miRs that are expressed in BMDMs but not in neuron-derived EVs.

**Fig. 2:**
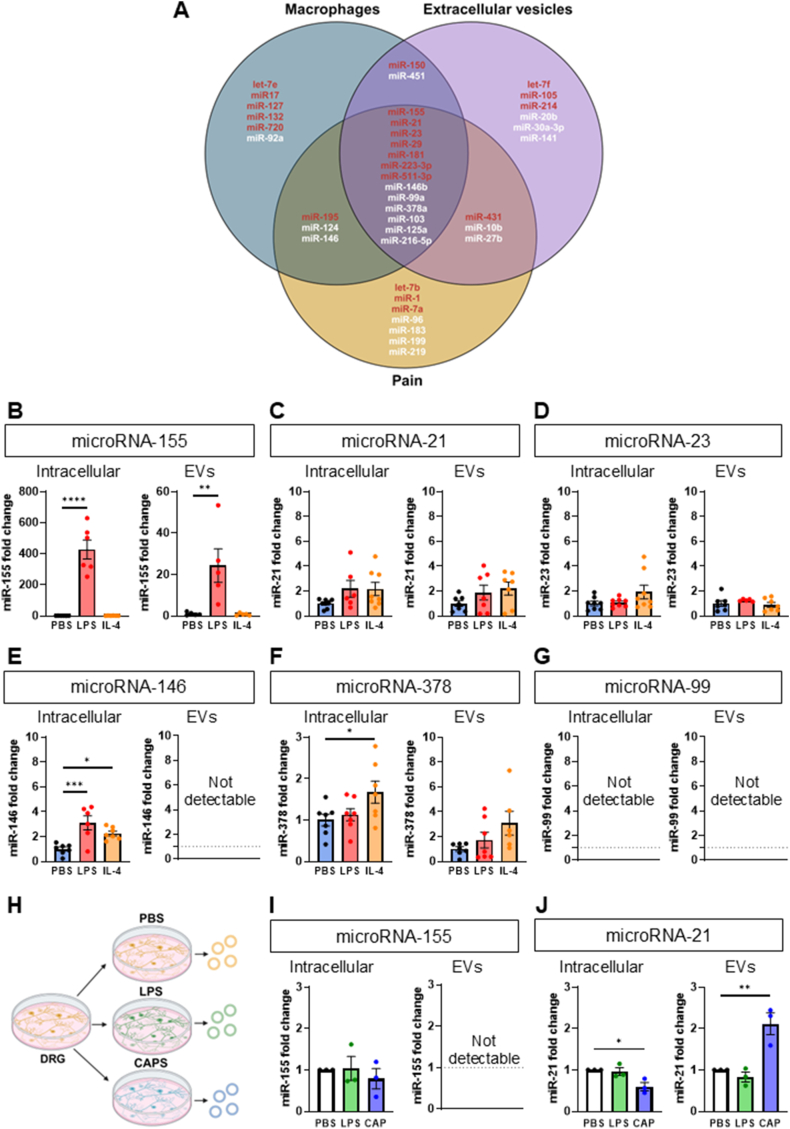
**Primary macrophages release extracellular vesicles that contain miR-155.** Data are presented as means ± SEM. ∗p < 0.05, ∗∗p < 0.01, ∗∗∗p < 0.001, ∗∗∗∗p < 0.0001 by One-way ANOVA with Dunnett’s (B-G) and Holm-Sidak (I,J) multiple-comparison test. **(A)** Venn Diagram showing microRNA selection process based on miRs expression in macrophages, in EVs and miRs association with nociception (red: pro-inflammatory, pro-nociceptive and disease associated; white: anti-inflammatory, anti-nociceptive and physiological/beneficial role in disease). **(B**–**G)** Quantification by RT-qPCR of miRs fold change over vehicle (PBS)-treated BMDMs and EVs compared to treatment with LPS (100 ng/ml) or IL-4 (20 ng/ml) for 16 h (n = 5–8 mice per group). **(H)** Schematic representation of dorsal root ganglia neuron (DRG) stimulation protocol with vehicle (PBS), LPS (100 ng/ml) or Capsaicin (CAP, 1 μM) for 3 h. **(I,J)** Quantification by RT-qPCR of miR-155 and miR-21 fold change over vehicle (PBS)-treated DRG compared to treatment with LPS (100 ng/ml) or Capsaicin (CAP,1 μM) for 3 h (n = 3 experiments per group. For each experiment DRG were pooled from 5 mice).

We mined currently available datasets [[12], [13], [14], [15], [16], [17], [18], [19], [20]] regarding miRs that are expressed by macrophages, contained in extracellular vesicles, and found to be dysregulated in models of neuropathic pain. We identified 13 candidates and selected six miRs, namely miR-155, miR-21, miR-378, miR-146, miR-99 and miR-23 (Fig. 2A). Our three inclusion criteria were as follows: miRs involved in macrophage polarisation, miRs identified in EVs and miRs associated with nociceptive mechanisms. We excluded 7 miRs for the following reasons: miR-29 and miR-103 were reported to have no selective effects on macrophage polarisation; miR-223, miR-125 and miR-181 were identified in macrophage cell lines other than in BMDMs; miR-511 and miR-216 were found in EVs isolated using protocols different from ultracentrifugation which is our preferred method.

Quantification of the six selected miRs in BMDMs showed miR-155 up-regulation both intracellular and in EVs after LPS, but not IL-4 treatment (∼440-fold intracellular and ∼22 fold in EVs) (Fig. 2B); expression of miR-21 and miR-23 was unchanged (Fig. 2C and D); miR-146 was only detected intracellular where expression increased by 3-fold and 2-fold following LPS and IL-4 polarisation, respectively (Fig. 2E); miR-378 increased intracellular (1.5-fold), but not in EVs, after IL-4 polarisation (Fig. 2F); miR-99 was detected in neither cells nor EVs (Fig. 2G). This data pointed to miR-155 as being a specific cargo component of M1-like BMDM-derived EVs. Such a specificity in macrophages was strengthened by comparison to cultured DRG neurons in which miR-155 intracellular content changed neither following LPS treatment (100 ng/ml) nor after capsaicin incubation (1 μM; selective activator of nociceptive neurons), and miR-155 was not detected in neuron-derived EVs (Fig. 2H and I). Instead, following capsaicin treatment, DRG neuron released EVs containing miR-21, as expected [5], and such a release was associated with lower miR-21 intracellular content (Fig. 2J).

This data suggests that EVs containing miR-155 are released by M1-like macrophages but not following noxious-like activation of DRG neurons. Therefore, we used miR-155 containing EVs to test the hypothesis that BMDM derived EVs are transferred to DRG neurons.

### 2.2 Macrophage-derived EVs are taken up by sensory neurons and transfer functional miR-155

With the aim to establish whether sensory neurons uptake macrophage-derived EVs, we exposed cultured DRG neurons to either EVs derived from LPS-BMDMs which contain miR-155 (miR-155^pos^EVs) or EVs derived from BMDMs transfected with miR-155 antagomir which were devoid of miR-155 (miR155^neg^ EVs).

**Fig. 3:**
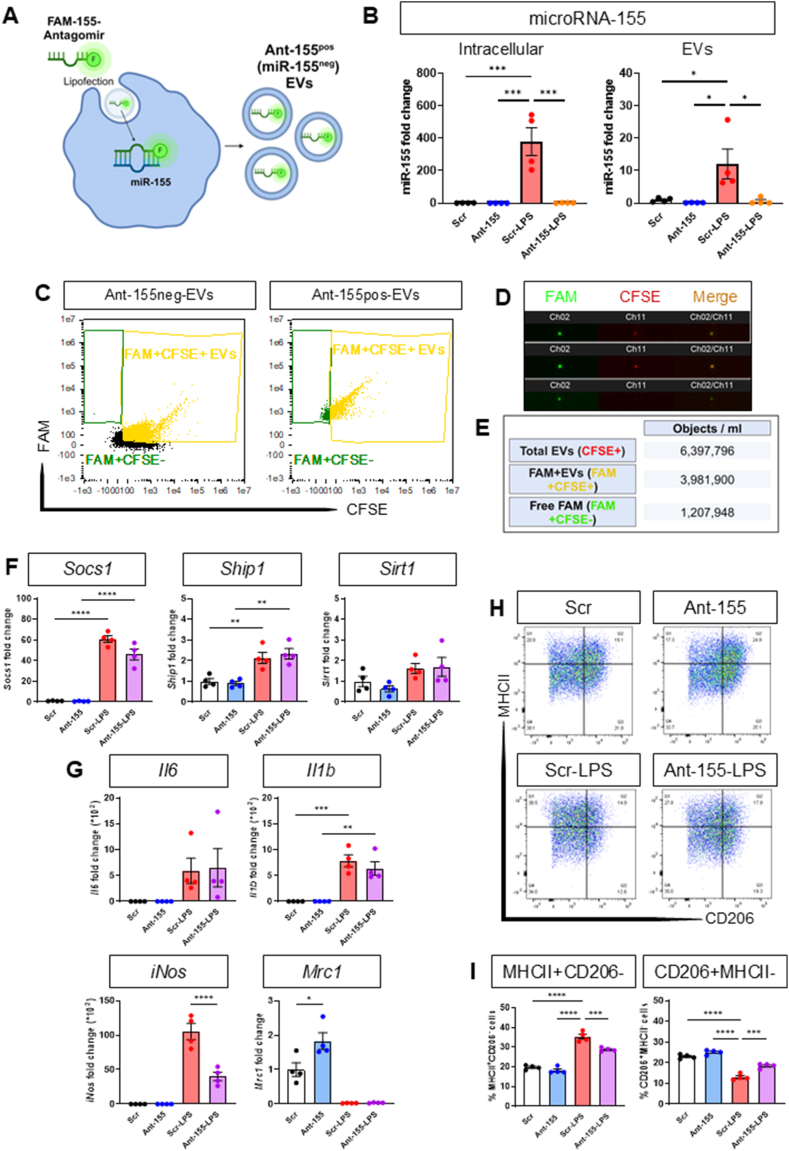
**BMDM transfection with miR-155 antagomir results in release of EVs devoid of miR-155 and reduction of M1-like macrophages.** Data are presented as means ± SEM. ∗p < 0.05, ∗∗p < 0.01, ∗∗∗p < 0.001, ∗∗∗∗p < 0.0001 by One-way ANOVA with Tukey’s multiple-comparison test. **(A)** Schematic representation of 48 h-transfection of BMDMs with either scrambled control (Scr) or miR-155 antagomir (Ant-155) followed by 16 h-treatment with vehicle or LPS (100 ng/ml (Scr-LPS or Ant-155-LPS). **(B)** Quantification by RT-qPCR of miR-155 fold change over scrambled control transfected BMDM intracellular and EVs compared to transfection with Ant-155 with or without LPS treatment (n = 4 mice per group). **(C)** Representative ImageStream^TM^ scatterplots of FAM expression in CFSE^+^ (BMDM-EVs) or CFSE^−^ events after miR-155 antagomir transfection. **(D)** Representative ImageStream^TM^ images of FAM-labelled antagomir transfected BMDM-EVs (Ch02 green) stained with CFSE (Ch11 red) (two upper panels) and free CFSE^−^ FAM-labelled antagomir (lower panel). **(E)** Table of ImageStream^TM^ quantification of Total-EVs (CFSE^+^), FAM^+^EVs (FAM^+^CFSE^+^) and free FAM (FAM^+^CFSE^−^) analysed as objects/ml after miR-155 antagomir transfection. **(F,G)** Quantification by RT-qPCR of *Ship1*, *Socs1*, *Sirt1*, *Il6*, *Il1b*, *iNos* or *Mrc1* fold change over scrambled control transfected BMDM intracellular compared to transfection with Ant-155 alone, LPS or Ant-155-LPS (n = 4 mice per group). **(H)** Representative flow cytometry scatterplots of MHCII and CD206 expression in CD11b^+^F4/80^+^ BMDMs at 48 h after miR-155 antagomir transfection. **(I)** Quantification of percentage of CD11b^+^F4/80^+^MHCII^+^CD206^-^ and CD11b^+^F4/80^+^CD206^+^MHCII^−^ cells at 48 h after miR-155 antagomir transfection (n = 4 mice per group).

To obtain miR-155^neg^ EVs, we transfected BMDMs with miR-155 antagomir (Fig. 3A) which resulted in inhibition of LPS-induced upregulation of miR-155 both intracellular (∼380-fold) and in EVs (∼11-fold) but no change in miR-155 basal levels (Fig. 3B). Furthermore, ImageStream^TM^ analysis of CFSE labelled EVs revealed presence of FAM-labelled miR-155 antagomir in EVs (3,961,900 objects/ml; 62 % of Total-EVs), most likely bound to miR-155, and revealed presence of naked (free/unbound) miR-155 antagomir (1,207,948 objects/ml) (Fig. 3C–E).

However, miR-155 antagomir transfection resulted in no change of intracellular levels of three miR-155 known targets, namely *Socs1*, *Ship1* and *Sirt1* neither under basal conditions nor after up-regulation induced by LPS (Fig. 3F). Similarly, *Il6* and *Il1b* up-regulation was not altered by miR-155 silencing. Nevertheless, after miR-155 silencing *iNos* decreased by about 4000-fold and *Mrc1* levels increased by about 2-fold (Fig. 3G). Additionally, using flow cytometry analysis we observed that miR-155 antagomir compared to scrambled transfection resulted in lower number of LPS-induced M1-like (MHCII^+^CD206^-^) macrophages and higher numbers of M2-like (CD206^+^MHCII^−^) macrophages (Fig. 3H and I).

**Fig. 4:**
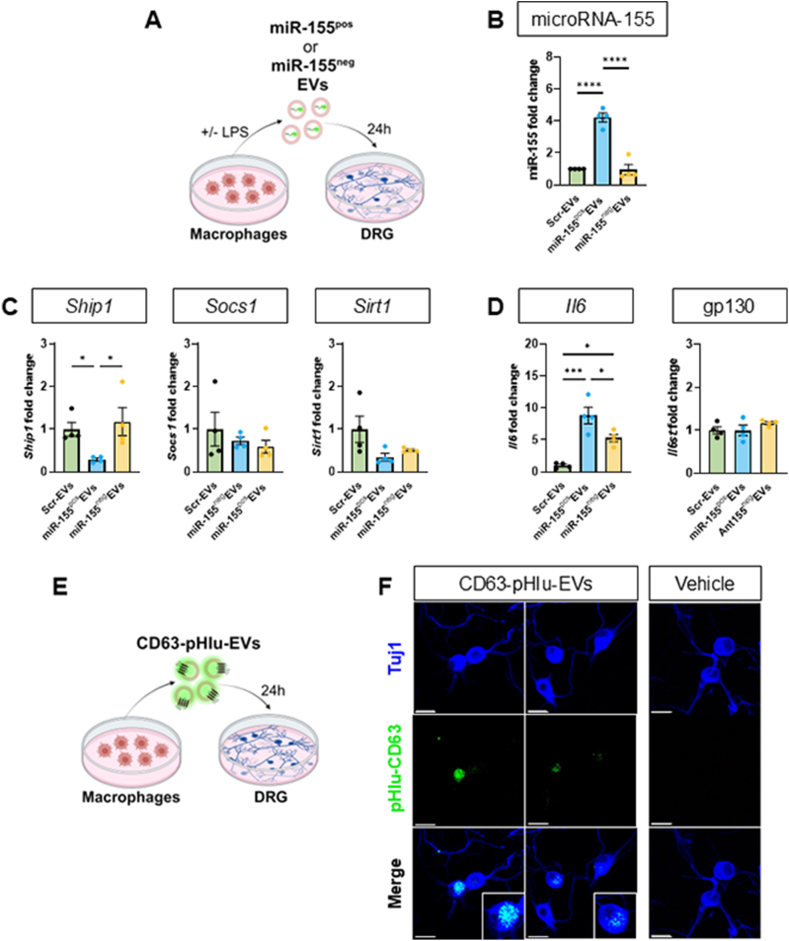
**Macrophage derived EVs are taken up by sensory neurons and transfer miR-155.** Data are presented as means ± SEM. ∗p < 0.05, ∗∗p < 0.01, ∗∗∗p < 0.001, ∗∗∗∗p < 0.0001 by One-way ANOVA with Tukey’s multiple-comparison test. **(A)** Schematic representation of DRG neurons 24 h-incubation with EVs (100 μg) derived from three types of BMDMs: i) transfected with scrambled control + 16 h PBS (Scr-EVs); ii) transfected with scrambled control + 16 h LPS (100 ng/ml, miR-155^pos^EVs) or iii) transfected with miR-155 antagomir + 16 h LPS (100 ng/ml, miR-155^neg^EVs). **(B**–**D)** RT-qPCR quantification of miR-155, *Ship1*, *Socs1*, *Sirt1*, *Il6* and *Il6st* (gp130) fold change over intracellular Scr-EV-incubated DRG compared to incubation with miR-155^pos^EVs and miR-155^neg^EVs (n = 4 mice per group). **(E)** Schematic representation of DRG neurons 24 h incubation with BMDM-derived CD63-pHIu-tagged EVs (100 μg). **(F)** Representative AiryScan confocal super-resolution images of DRG neurons (Tuj1: blue) incubated with EVs derived from BMDMs transfected with pHIu-CD63-EVs (green) and treated with vehicle (PBS) for 24 h. Scale bar: 20 μm.

Overall, such an *in-vitro* approach provided us with BMDM-derived EVs containing miR-155 and EVs devoid of miR-155 that also carried miR-155 antagomir. We then used these EVs to evaluate whether DRG neurons uptake BMDM-derived EVs and if such uptake could repress known miR-155 targets. We observed that exposure of DRG neurons to miR-155^pos^ EVs, but not miR-155^neg^ EVs, resulted in a 4-fold increase of miR-155 content (Fig. 4A and B), downregulation of miR-155 target gene *Ship1* (∼0.4-fold) but not *Socs1* and *Sirt1* (Fig. 4C) and up-regulation of indirect target *Il6* (∼9-fold) but no change in IL-6 receptor gp130 (*Il6st*) expression (Fig. 4D). Thus, we obtained successful transfer of functional miR-155 from BMDM-derived EVs to sensory neurons. This uptake was further confirmed by the observation that BMDM-derived vesicles tagged with an EV-specific encodable fluorescent protein, CD63-pHIu [21] readily accumulated in cultured DRG neurons (Fig. 4E and F).

**Fig. 5:**
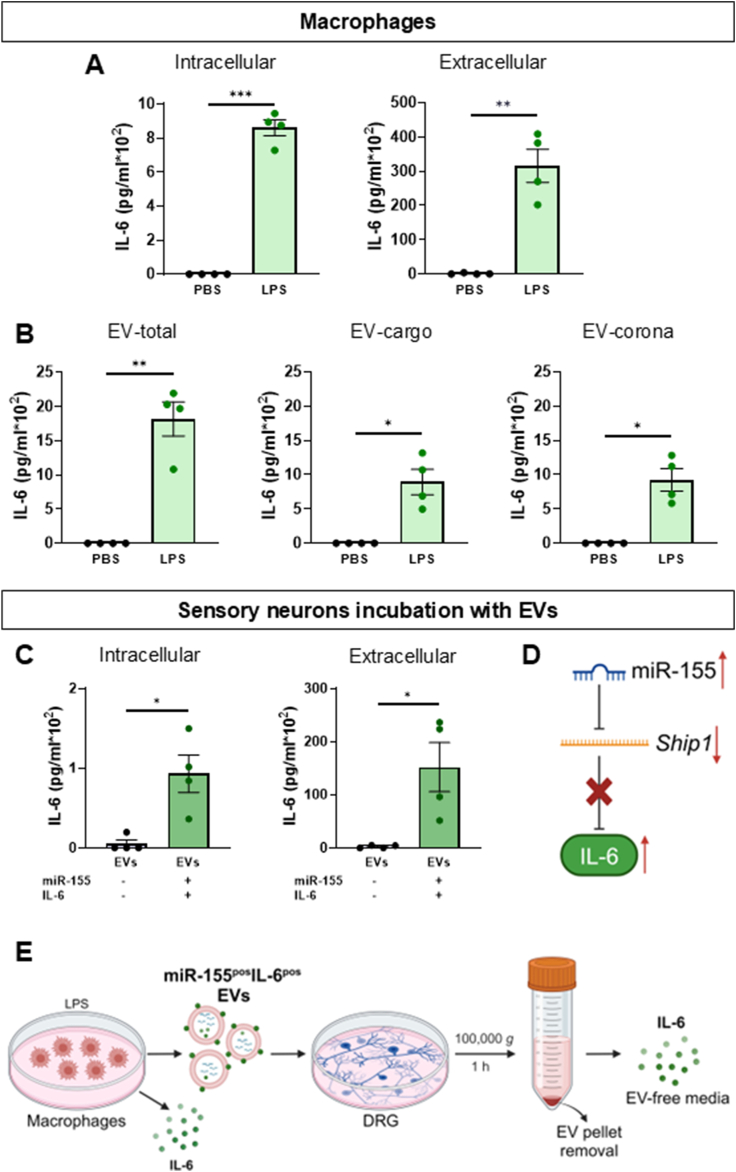
**BMDMs transfer IL-6 to DRG neurons via miR-155**^**pos**^**IL6**^**pos**^**EVs.** Data are presented as means ± SEM. ∗p < 0.05, ∗∗p < 0.01, ∗∗∗p < 0.001, ∗∗∗∗p < 0.0001 by paired (A,B) or unpaired (C) two-tailed Student’s *t*-test. **(A,B)** IL-6 protein levels (pg/ml) in BMDMs: intracellular, extracellular (in culture media), EV-total, EV-cargo and EV-corona content after either vehicle (PBS) or LPS stimulation (100 ng/ml, 16 h) (n = 4 mice per group). **(C)** IL-6 protein levels (pg/ml) in DRG sensory neurons: intracellular and extracellular (in culture media) after incubation of either miR-155^neg^IL6^neg^BMDM-EVs or miR-155^pos^IL6^pos^BMDM-EVs (100 μg, 24 h) (n = 4 mice per group). **(D)** Schematics of miR-155-suppression of *Ship1*-mediated inhibition of IL-6 expression. **(E)** Schematic illustration of IL-6 location (green dots) in macrophages after LPS treatment and in DRG neurons after incubation with miR-155^pos^IL6^pos^BMDM-EVs.

We became intrigued by *Il6* increase in DRG neurons following transfer of miR-155^pos^ EVs because, although IL-6 is not a direct miR-155 target, it is a pro-nociceptive cytokine that binds to the transmembrane protein gp130 to promote nociceptive signalling in sensory neurons [22,23]. Thus, we quantified intracellular and extracellular IL-6 levels in BMDMs and observed that, as expected, LPS treatment increased IL-6 intracellular content and extracellular release in the culture media compared to controls (Fig. 5A). In addition, whilst control EVs were devoid of IL-6, LPS induced release of EVs (EV-total) containing IL-6 inside the vesicle (EV-cargo) and membrane bound (EV-corona) (Fig. 5B). When we incubated miR-155^pos^IL6^pos^ EVs with cultured DRG neurons, which normally express very low levels of IL-6, we measured higher IL-6 content compared to incubation with IL-6^neg^ EVs both intracellular (∼100 pg/ml) and extracellular in DRG media (∼15,000 pg/ml) compared to incubation with IL6^neg^ EVs (Fig. 5C). This data suggests that miR-155^pos^ EVs mediate IL-6 transfer to neurons which is partially explained by handover of EV-total IL-6 (∼1700 pg/ml). However, for extracellular IL-6 in neuron culture media to reach 15,000 pg/ml, it is plausible to suggest that EV transfer of miR-155 also contributed to IL-6 up-regulation, possibly through suppression of *Ship1*-mediated inhibition of IL-6 expression [24] (Fig. 5D).

Data obtained thus far provided *in-vitro* evidence that BMDM-derived EVs are taken up by DRG neurons and deliver functional cargos (Fig. 5E) and that miR-155 is one of the key active elements of these cargos. To probe potential *in-vivo* relevance of macrophage communication with sensory neurons under neuropathic pain conditions, we tested whether silencing of miR-155 expression in DRG resulted in attenuation of neuropathic allodynia.

### 2.3 Intrathecal delivery of macrophage-derived EVs containing miR-155 antagomir attenuates neuropathic allodynia

**Fig. 6:**
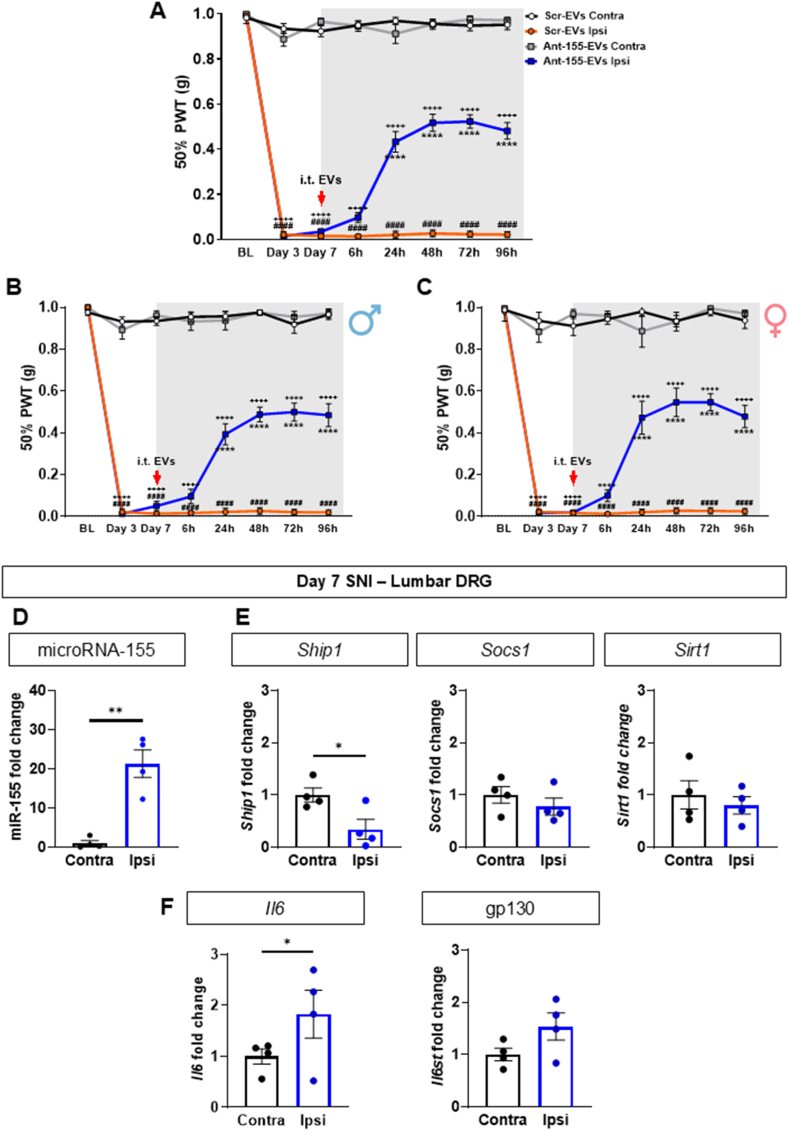
**Intrathecal delivery of macrophage-derived EVs containing miR-155 antagomir attenuates neuropathic allodynia**. Data are presented as means ± SEM. ∗p < 0.05, ∗∗p < 0.01, ∗∗∗p < 0.001, ∗∗∗∗p < 0.0001 by Two-way ANOVA with Tukey’s multiple-comparison test (A-C) and paired two-tailed Student’s *t*-test (D-F). **(A**–**C)** Effect of i.t. injection of scrambled control-transfected BMDM-derived-EVs (Scr-EVs) and miR-155 antagomir-transfected BMDM-derived-EVs (Ant-155-EVs) (2 μg) on SNI mechanical hypersensitivity of pulled cohort (A), male (B) and female (C) mice. Contralateral uninjured (Contra) or ipsilateral injured (Ipsi) thresholds are presented as 50 % paw withdrawal threshold (PWT, g) (n = 16 mice per group, 8 males and 8 females). **(D**–**F)** RT-qPCR quantification of miR-155, *Ship1*, *Socs1*, *Sirt1*, *Il6* and *Il6st (*gp130) fold change in day 7 after spared nerve injury mice (SNI) lumbar DRG over contralateral (uninjured, contra) compared to ipsilateral (injured, Ipsi) (n = 4 mice per group).

**Fig. 7:**
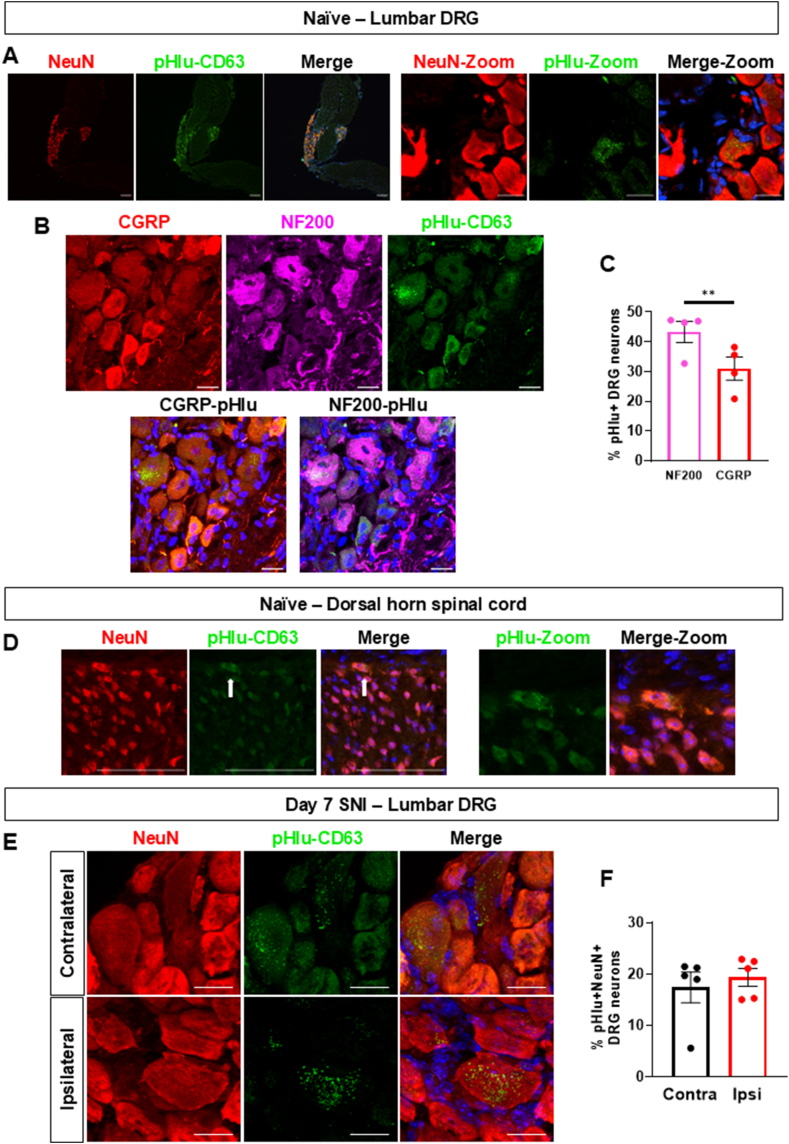
**Intrathecally delivered macrophage-derived EVs accumulate in DRG neurons** Data are presented as means ± SEM. ∗p < 0.05, ∗∗p < 0.01, ∗∗∗p < 0.001, ∗∗∗∗p < 0.0001 by paired two-tailed Student’s *t*-test. **(A)** NSPARC super resolution confocal images of lumbar DRG neurons (NeuN: red) at 2 h after i.t. injection of CD63-pHIu-EVs (green, 100 μg). Whole DRG at 10x magnification (scale bar: 150 μm) (left panel) and 60x magnification (scale bar: 20 μm) (right panel). **(B)** Representative confocal images of lumbar DRG (CGRP: red or NF200: magenta) sections at 2 h after i.t. injection of CD63-pHIu-EVs (green, 100 μg) (scale bar: 20 μm) **(C)** Quantification of pHIu-CD63^+^NF200^+^ and pHIu-CD63^+^CGRP^+^ sensory neurons represented as percentage (%) (n = 4 mice per group, L4 DRG with 3 images per mouse and 30 neurons per condition). **(D)** Representative confocal images of lumbar spinal cord laminae I-II-III (NeuN: red) sections at 2 h after i.t. injection of CD63-pHIu-EVs (green, 100 μg) (scale bar: 150 μm) **(E)** Representative confocal images of contralateral and ipsilateral day 7 SNI lumbar DRG (NeuN: red) sections 2 h after i.t. injection of CD63-pHIu-EVs (green, 100 μg) (scale bar: 20 μm) **(F)** Quantification of pHIu-CD63^+^NeuN^+^ contralateral and ipsilateral day 7 SNI sensory neurons represented as percentage (%) (n = 5 mice per group, L4 DRG with 3 images per mouse and 40 neurons per condition).

**Fig. 8:**
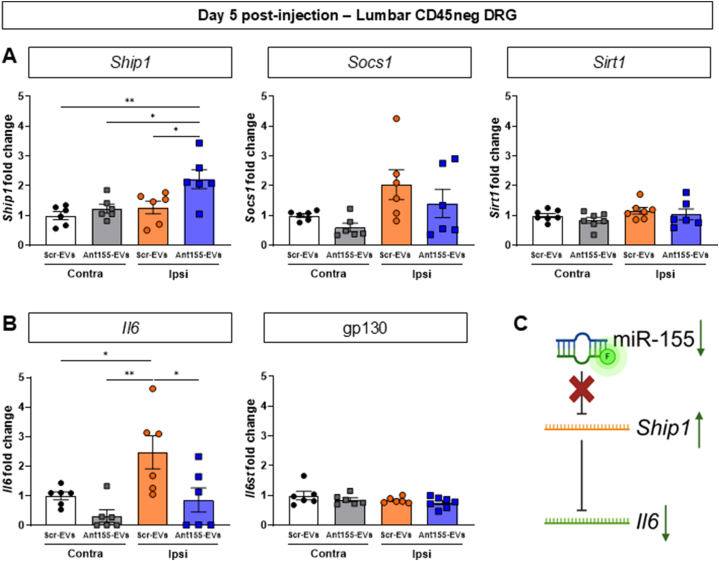
**Intrathecal delivery of macrophage-derived EVs containing miR-155 antagomir increases *Ship1* and decreases *Il6* expression in sensory neurons.** Data are presented as means ± SEM. ∗p < 0.05, ∗∗p < 0.01, ∗∗∗p < 0.001, ∗∗∗∗p < 0.0001 by One-way ANOVA with Tukey’s multiple-comparison test. **(A,B)** RT-qPCR quantification of *Ship1*, *Socs1*, *Sirt1*, *Il6* and *Il6st (*gp130) fold change over contralateral (Contra) miR-155-antagomir^neg^ EVs (Scr-EVs) injected CD45neg DRG cells compared to contralateral or ipsilateral (Ipsi) miR-155-antagomir^pos^ EVs (Ant155-EVs) injected (2 μg) (n = 6 mice per group, each sample was obtained from a pool of 3 lumbar DRG/mouse from 4 mice). **(C)** Schematics of miR-155 antagomir-suppression of miR155-mediated disinhibition of *Il6* expression.

We confirmed that peripheral nerve injury (SNI) was associated with development of mechanical hypersensitivity (Fig. 6A) and observed that intrathecal (i.t.) delivery of macrophage-derived EVs containing miR-155 antagomir resulted in 50 % reversal of SNI mechanical hypersensitivity at 24 h after injection (Fig. 6A). Such an anti-allodynic effect was maintained for up to 4 days in male and female mice (Fig. 6B and C). Furthermore, we found that miR-155 expression was detectable in uninjured DRG contralateral to SNI and it was up-regulated by 20-folds in ipsilateral DRG at day 7 SNI (Fig. 6D) alongside downregulation of direct target *Ship1* and up-regulation of indirect target *Il6* (Fig. 6E and F). This data suggested the possibility that intrathecal injected EVs had delivered functional miR-155 antagomir to the DRG that we tested by injecting CD63-pHIu-tagged EVs. We observed that, in naïve mice, intrathecal EVs readily reached lumbar DRG at 2 h after injection and accumulated in neurons (NeuN expression) (Fig. 7A) including large (NF200+) and small (CGRP+) cell bodies (Fig. 7B), though EVs preferentially gathered in NF200+ neurons (Fig. 7C). Little to none EVs were detected in the dorsal horn of the spinal cord (Fig. 7D). In day 7 SNI mice, we confirmed that intrathecal EVs reached the lumbar DRG and observed vesicles distribute equally between contralateral uninjured DRG and ipsilateral injured DRG (Fig. 7E and F). Furthermore, gene expression analysis of DRG cells excluding leukocytes (CD45neg population) obtained from ipsilateral and contralateral lumbar DRG at day 5 after intrathecal injection of EVs revealed increased *Ship1* expression (∼2-fold, Fig. 8A) and downregulation of *Il6* (∼1-fold, Fig. 8B). Overall, this data suggests that accumulation of EVs containing miR-155 antagomir in sensory neurons inhibited production of pro-nociceptive cytokine *Il6* which may occur via disinhibition of *Ship1* a direct miR-155 target (Fig. 8C) and this pathway underlies the anti-allodynic effect of BMDM derived EVs.

## 3 Discussion

The major finding of this work is threefold: i) macrophage-derived EVs injected intrathecal preferentially target DRG neurons cell bodies; ii) miR-155 is an active component of M1-like macrophages; iii) intrathecal delivery of macrophage-derived EVs containing miR-155 antagomir attenuates neuropathic hypersensitivity. Our evidence indicates that EV-mediated delivery of miR-155 antagomir results in disinhibition of a known miR-155 target, namely *Ship1*, in DRG neurons (CD45neg population) and down-regulation of pro-nociceptive cytokine *Il6*. It is plausible to speculate that disinhibition of SHIP1-mediated inhibition of STAT, AKT and NF-kB, results in up-regulation of IL-6 expression in neurons [25].

Our work is a novel addition to the concept that DRG sensory neurons and macrophages (and potentially other cell types within the peripheral somatosensory system) communicate through EV-exchange following peripheral nerve injury. Specifically, this study identifies sensory neuron cell bodies as receivers of a specific macrophage cargo including a microRNA, miR-155, and a cytokine, IL-6. We do not yet provide an explanation for EV preferential accumulation in DRG over the dorsal horn of the spinal cord. However, it is possible that EVs encounter more barriers that impair penetrability in the spinal cord and if delivered in higher amounts, some EVs would enter the spinal cord. A similar argument would apply to the observation that not all DRG neurons taken up EVs: larger amounts of EVs would make more EVs available for neuron uptake and help figure out whether the small 10 % difference in EV accumulation between large and small size neurons reaches biological relevant values of at least 30 %.

As one of the pro-inflammatory microRNAs, miR-155 is involved in several immune processes such as macrophage polarisation to M1-like phenotype [15]. Indeed, we found that miR-155 is a cargo component of EVs released by M1-like macrophages that is transferred to cultured DRG neurons where miR-155 results in suppression of *Ship1* expression and increase of IL-6 expression. Since, satellite cells are present with neurons in cultured DRG preparations and express both IL-6 and TLR4 [26,27], we cannot rule out that they uptake EVs and release IL-6 after TLR4 receptor activation. Furthermore, although miR-155 targets also include *Socs1* and *Sirt1*, we could not detect any changes in such genes possibly because of lack of sensitivity of our assay. For instance, sensory neurons express high levels of *Socs1* [28] which might require delivery of more miR-155 to reveal down-regulation of expression.

Notably, miR-155 direct and indirect target, namely *Ship1* and IL-6, are steadily regulated under *in-vitro* and *in-vivo* conditions and suggest the intriguing possibility that following nerve injury, miR-155 transfer by macrophages contributes to IL-6 expression in sensory neurons.

Indeed, IL-6 is upregulated in DRG as soon as 24 h after nerve injury because Schwann cells at the site of injury release CNTF that promotes phosphorylation and up-regulation of STAT3 and pSTAT-3 is retrogradely transported to cell bodies where it up-regulates IL-6 [29,30]. In addition, we propose that pro-inflammatory macrophage-mediated transfer of EV-containing miR-155 to neurons constitutes a local DRG mechanism for IL-6 up-regulation after axonal injury, although we cannot exclude that other factors regulate such a cytokine expression. Neuronal activity would then result in IL-6 release and consequent activation of neuron-expressed gp130 that facilitates nociceptive transmission towards the dorsal horn of the spinal cord on its way to the higher centres. IL-6 can induce signalling in cells via either classical receptor signalling by binding to membrane bound complex gp130/IL-6Ra or via trans-signalling pathway by binding to soluble IL-6Ra and then membrane bound gp130. Membrane bound IL-6Ra is expressed by immune cells and not expressed in DRG neurons [31]. However, IL-6 soluble receptor levels increase after nerve injury and this increase is likely mediated by release from satellite glial cell [31]. Gp130 is expressed in DRG neurons and gp130 silencing in nociceptive neurons (Na_v_1.8 expressing small-diameter neurons) is associated with reduced mechanical allodynia in models of chronic pain [32].

Our study entails a potential antinociceptive translatability for EV- and microRNA-based approaches. Therapies based on microRNAs are promising as miR levels can be regulated using mimics or inhibitors. The main disadvantage of microRNA-therapy are possible off-target effects. However, targeted delivery to a specific cell type by encapsulating miRs into EVs can offer a practical solution.

## 4 Limitations of the study

We delivered miR-155 antagomir encapsulated in macrophage-derived vesicles where some antagomir would be bound to endogenous miR-155. Although we have evidence that our EVs also contained naked antagomir, we cannot establish with certainty how much free antagomir was delivered. The use of nanoparticles encapsulating miR-155 antagomir is a potential alternative for a more controlled delivery.

## 5 Methods

### 5.1 Animals

Studies were conducted in 8–12-week-old C57BL/6J male and female mice (in-house breeding) under ethical approval from local animal guidance for care and use of laboratory animals, and in accordance with the UK Home Office regulations (Guidance on the Operation of Animals, Scientific Procedures Act, 1986; Project Licences PCD4DEDAC9 and PP4092047). Mice received food and water *ad libitum* and were kept at room temperature with a 12h-light/dark cycle housed in groups of maximum 5 mice.

### 5.2 Bone marrow derived macrophages (BMDMs) in culture

BMDMs were isolated by flushing bone marrows from femurs and tibias of adult mice and differentiated for 7 days at 37 °C and 5 % CO_2_ in Dulbecco’s Modified Eagle’s medium (DMEM, Gibco, Cat#21063-029) supplemented with 10 % (v/v) heat-inactivated foetal bovine serum (HI FBS, Gibco), 1 % penicillin/streptomycin (P/S, Thermo Fisher Scientific, Cat#15140-122) and 10 % L929 cell-conditioned medium as a source of macrophage colony stimulating factor. On day 7, cells were detached and plated in DMEM supplemented with 1 % HI (heat inactivated) exosome-depleted FBS and 1 % P/S. Subsequently, macrophages were incubated for classical polarisation protocols with either lipopolysaccharide (LPS, 100 ng/ml, Sigma Aldrich, Cat#L2630) or interleukin-4 (IL-4, 20 ng/ml, Bio-Techne, Cat#404-ML) for 16 h. For NanoSight^TM^, RT-qPCR and Flow Cytometry cells were plated at 1x10^6^ cells/well density and for Western blotting at 2x10^6^ cells/well density.

### 5.3 Dorsal root ganglia (DRG) in culture

DRG from adult mice were collected and dissociated using dispase (3 mg/ml, Roche, Cat#04942078001) and collagenase (0.1 %, Scientific Laboratory Supplies, Cat#C9407) in Hank’s Balanced Salt Solution (HBSS, Gibco, Cat#14170-088). DRG were then triturated by pipetting, and cell suspensions centrifuged at 900 rpm for 5 min. Pellets were resuspended in neurobasal medium (1X, Gibco, Cat#21103-049) supplemented with GlutaMAX^TM^ (100X, 1 %, Gibco, Cat#35050-061), penicillin/streptomycin (1 %) and B27 supplement (exosome free, 2 %, Fisher Scientific UK, Cat#17504-044) to improve neuron survival, and plated on poly-L-lysine (0.1 %, Cat#P6282) and laminin (40 μg/ml, Scientific Laboratory Supplies, Cat#L2020) pre-coated plates. DRG neurons were cultured at 20,000 cells/well density and incubated at 37 °C and 5 % CO_2_ for 24 h before treatment with LPS (100 ng/ml) or capsaicin (1 μM, Sigma Aldrich, Cat#M2028) for 3 h or incubation with BMDM-derived EVs (100 μg) for 24 h. Satellite cell proliferation was not a concern because cells were cultured for 24h and proliferation occurs in longer term cultures.

### 5.4 Isolation and nanoparticle tracking analysis (NTA) of extracellular vesicles (EVs)

EVs from BMDM media were isolated via differential ultracentrifugation. Media were centrifuged at 4,000 *g* for 15 min at 4 °C to remove cells and cell debris and then at 13,000 *g* for 5 min at 4 °C to remove apoptotic bodies. Supernatants were further centrifuged at 100,000 *g* for 1 h at 4 °C to isolate EVs. EV pellets were resuspended in double filtrated (0.22 μm filters) phosphate-buffered saline (PBS, Sigma Aldrich, Cat#D8537) and subjected to size and concentration analysis with an NS300 Nanoparticle tracker with 488 nm scatter laser and high-sensitivity camera (Malvern Instruments Ltd., UK). Data analysis was conducted using the NTA2.1 software (NanoSight^TM^, Malvern, UK) with the following settings: camera level: 14, detection threshold: 5, Blur: auto and minimum expected particle size: 20 nm.

### 5.5 Transmission electron microscopy (TEM) of EVs

EV suspension was added to formvar coated TEM grids and after 30 min fixed with 1 % glutaraldehyde in PBS. Subsequently, the grids were stained with 0.4 % uranyl acetate in 2 % methyl cellulose solution. 80,000x magnification images were acquired using a JEOL 1400+ TEM (JEOL ltd, Tokyo, Japan) equipped with an AMT NanoSprint 12 camera (AMT Imaging Direct, Woburn, MA, USA).

### 5.6 Western blot of BMDMs and EVs

BMDMs and EVs were lysed in RIPA buffer (Fisher Scientific UK, Cat#89900) supplemented with Halt^TM^ protease and phosphatase inhibitor cocktail (1:100, Fisher Scientific UK, Cat#1861281). Samples were sonicated and protein concentration quantified with BCA^TM^ Protein Assay kit or Micro BCA^TM^ Protein Assay kit (Fisher Scientific UK, Cat#23227 and Cat#23235) for cells and EV fractions, respectively. Samples were subsequently resuspended in Laemmli sample buffer (Bio-Rad) and heated for 15 min before being loaded into 10 % w/v sodium dodecyl sulphate polyacrylamide tris–glycine gel (10 % MP TGX Gel 10W, Bio-Rad Laboratories, Cat#4561033) and transferred into polyvinylidene difluoride (PVDF) membranes using wet tank 25 V overnight transfer method. Membranes were blocked with 5 % non-fat-dried milk in TBS-Tween (50 mM Tris-HCl, pH 7.6, 150 mM NaCl, 0.1 % Tween 20, Cat#P2287) for 1 h at room temperature (RT) and followed by overnight incubation with primary antibodies: rabbit anti-TSG101 (1:1000, Sigma-Aldrich, Cat#AV38773), rabbit anti-CD63 (1:1000, Abcam, Cat#ab217345), rabbit anti-ANNEXIN-A1 (1:1000, Abcam, Cat#ab214486) and rabbit anti-CALNEXIN (1:1000, Abcam, Cat#ab22595). Protein levels were quantified by densitometry scanning using Fiji (ImageJ, 1.52i, Wayne Rasband, USA) and analysed as ratio EVs/cells of origin.

### 5.7 Real-time quantitative PCR (RT-qPCR)

Total and small RNA were isolated using miRVana^TM^ miRNA Isolation Kit (Life Technologies, Cat#AM1561). RNA concentration and purity were measured using the NanoDrop ND-100 Spectrophotometer (Labtech). For total RNA detection, 100 ng of RNA were reverse transcribed using QuantiTec Reverse Transcription Kit (Qiagen, Cat#205311). For microRNA detection, 30 ng of small RNA were reverse transcribed with miRCURY LNA^TM^ RT Kit (Qiagen, Cat#339340). For real-time PCR, Lightcycler 480 Sybr Green I Master (Roche, Cat#04707516001) and the following specific mouse genes primers in a LightCycler 480 (Roche) were used: Mm_*Nos2*_1_SG (Qiagen, Cat#QT00100275), Mm_*Il6*_1_SG (Qiagen,Cat#QT00098875), Mm_*Il1b*_2_SG (Qiagen, Cat#QT01048355), *Tnfa* (Sigma Aldrich, Cat#SY190615463-095), *Mrc1* (Sigma Aldrich, Cat#SY190608845-031), *Arg1* (Sigma Aldrich, Cat#SY190608845-017), Mm_*Inpp5*_1_SG (*Ship1*, Qiagen, Cat#QT00116655), Mm_*Socs1*_1_SG (Qiagen, Cat#QT01059268), Mm_*Sirt1*_2_SG (Qiagen, Cat#QT01055642), Mm_*Il6st*_1_SG (gp130, Qiagen, Cat#QT00106456), Mmu-miR-155-5p, Mmu-miR-21-5p, Mmu-miR-23a-5p, Mmu-miR-146b-3p, Mmu-miR-378a-3p, Mmu-miR-99a-3p miRCURY LNA miRNA PCR Assays (Qiagen, Cat#YP02119303, Cat#YP00204230, Cat#YP00205631, Cat#YP00205323, Cat#YP00204179, Cat#YP02106991, Cat#339146YCI0204024). The relative expression of RNA was calculated by the 2^−ΔΔCT^ method and normalized to *Hprt1* (Qiagen, Cat#QT00059066) as housekeeping gene for mRNA and *Unisp6* spike-in control for miRNA.

### 5.8 Transfection of BMDMs

Lipofectamine^TM^ 3000 (Fisher Scientific UK, Cat#L3000015) was used to transfect FAM-labelled miR-155-5p antagomir (miRCURY LNA^TM^ miRNA Custom Power Inhibitor (5) – MMU-MIR-155-5P CUSTOM MIRCURY:/56-FAM/CCCCTATCACAATTAGCATT, Qiagen, Cat#339146YCI0204024), scrambled-control or pCMV-Sport6-CD63-pHIourin (CD63-pHIu [21], Addgene plasmid #13090) plasmid (1 μg) in macrophages using the reverse transfection method. Transfected cells were then cultured for 48 h at 37 °C and 5 % CO_2_ before usage. For FAM-miR-155-5p antagomir transfection, efficiency of 86.5 % was validated with flow cytometry using F4/80, CD11b and FAM-antagomir label as markers (data not shown). For CD63-pHIu plasmid transfection, efficiency of 79.82 % was validated by double positive staining for pHIu and anti-GFP antibody (data not shown).

### 5.9 ImageStream^TM^ analysis of EVs

Culture media were centrifuged to remove cells and cell debris as previously described. Supernatants were then incubated for 20 min at 37 °C with CellTrace far-red dye (CFSE, 1 μM, Fisher Scientific UK, Cat# C3456) followed by 100,000 *g* ultracentrifugation for 1 h at 4 °C to isolate EVs. ImageStream^TM^ analysis of EVs was performed as previously described [5,10] with 658 nm laser set at 200 mW and side scatter at 70 mW with 60x magnification. Data were analysed with IDEAS software (Amnis, EMD Millipore).

### 5.10 Flow cytometry of BMDMs

BMDMs were washed with ice-cold PBS, scraped and centrifuged at 350 g for 5 min. Samples were stained for viability using Live/Dead Fixable Near IR (Invitrogen, Cat#L10119) in PBS for 30 min, then stained with a mix of directly conjugated antibodies in FACS buffer (0.5 % BSA and 2 mM EDTA in PBS). We used anti–mouse CD16/CD32 (clone 93, BioLegend, Cat#101302) for blocking and the following antibody panel: BV421-conjugated anti-CD11b (clone M1/70, Biolegend UK, Cat#101235), APC-conjugated anti-F4/80 (clone BM8, Biolegend UK, Cat#123116), PE-Cy7–conjugated anti-CD206 (clone C068C2, Biolegend UK, Cat#141720), PercP-Cy5.5–conjugated anti-MHCII (clone AF6-120.1, Biolegend UK, Cat#116416) (1:100). Cells were analysed with LSRII-Fortessa^TM^ flow cytometer (BD Biosciences) and FlowJo^TM^ software (V10.7.2, BD Biosciences). Events were gated as CD11b^+^F480^+^, CD11b^+^F4/80^+^MHCII^+^CD206^–^ and CD11b^+^ F4/80^+^CD206^+^MHCII^–^ to identify total, M1-like and M2-like macrophages, respectively.

### 5.11 Immunofluorescence of spinal cord and DRG *in-vitro* and *in-vivo*

DRG cultures were incubated for 24 h with EVs (100 μg) derived from CD63-pHIu-trasfected macrophages with or without LPS stimulation as previously described. Following incubation, DRG were fixed with ice-cold 4 % paraformaldehyde (PFA, Sigma-Aldrich, Cat#SC281692) for 20 min and permeabilized/blocked for 1 h at RT in blocking buffer (0.05 % Tween-20, 0.25 % Triton-X-100 and 5 % donkey serum in PBS).

For lumbar 3-4-5 DRG and spinal cord tissue immunostaining, mice were anaesthetised with an intraperitoneal injection of pentobarbital (Pentoject) and perfused with ice-cold PBS. DRG and spinal cord were quickly extracted and placed in ice-cold 4 % PFA for 4 h followed by overnight incubation at 4 °C in 30 % sucrose solution. DRG and spinal cords were then sectioned with a cryostat (Bright Instruments, 10–20 μm). Tissue sections were permeabilised with PBS-T (0.1 % Triton X-100 in PBS) for 10 min and blocked in TBS-T (0.25 % Triton X-100, 0.05 % Tween-20 in PBS) and 5 % BSA for 1 h.

Spinal cord and DRG cultures or sections were incubated overnight at 4 °C with rabbit anti-mouse b-III-tubulin (Tuj1, 1:1000, Abcam, Cat#ab18207), rabbit anti-mouse NeuN (1:750, Cell signaling, Cat#08/201512943S), goat anti-mouse CGRP (1:500, Abcam, Cat#AB36001) or mouse anti-mouse NF200 (1:400, Sigma Aldrich, Cat#N0142-0000142491) followed by incubation with secondary antibodies Alexa Fluor 405, 546 or 680 (Invitrogen, 1:1000). The immunoreactivity was captured using AiryScan super resolution detector in ZEISS LSM 800 series confocal microscope (Bio-imaging and Flow Cytometry Facility University of Leeds) for DRG cultures and Zeiss LSM 400 (LSM software) confocal microscope at 63X oil or NSPARC detector in Ti2 Ax System confocal microscope (Nikon Imaging Centre, KCL) for DRG and spinal cord sections.

### 5.12 Enzyme-linked immunosorbent assay (ELISA)

IL-6 levels (pg/ml) were quantified using an enzyme-linked immunoassay kit (Mouse IL-6 Uncoated ELISA, Invitrogen, Cat#88–7064) following manufacturer’s instructions.

### 5.13 Intrathecal injections

Under light isoflurane anaesthesia, intrathecal injections (5 μl/mouse) between lumbar 4 and 5 vertebrae were performed using a 30 G needle.

### 5.14 Behavioural testing

Static mechanical withdrawal thresholds were assessed by applying calibrated von Frey monofilaments (0.008–1.0 g) to the plantar hind paw. Mice were placed in individual compartments, and all tests began after 30 min habituation during the light cycle. 50 % paw withdrawal threshold (PWT) was determined by increasing or decreasing stimulus intensity and evaluated using Dixon’s method as previously described [5,10]. Testing started with the application of a 0.07 g filament and each paw was assessed alternately between application of increasing stimulus intensity until a withdrawal response was achieved or application of 1.0 g filament failed to induce a response. The experimenter was blinded to treatment groups.

### 5.15 Isolation of CD45^negative^ cell population from lumbar DRG

DRG digested in a single cell suspension were passed through MS MACS columns (Miltenyi Biote, Cat#130-042-401) placed on a magnetic field separator after labelling with anti-CD45 MicroBeads (Miltenyi Biotec, Cat#130-052-3015240103085) for 15 min at 4 °C. CD45^negative^ unlabelled cells (negative fraction) were collected and centrifuged at 300 *g* for 10 min. Pellets were then lysed for RNA extraction.

### 5.16 Induction of peripheral neuropathy

Spared nerve injury (SNI) model was performed as previously described [5,10]. Briefly, the three terminal branches of the sciatic nerve were exposed under isoflurane anaesthesia (2.5 %) by a small incision in the skin and blunt incision of the left tight. The sural nerve was identified and left intact while the peroneal and tibial nerves were cut, and the distal nerve stump removed.

## 6 Quantification and Statistical analysis

Statistical analysis was performed using GraphPad Prism Software. Data are expressed as means ± SEM and analysed with Two-tailed paired or unpaired student’s *t*-test (2 groups), One-way ANOVA with Dunnett’s, Tukey’s or Holm-Sidak’s multiple-comparison test (3 or more groups) or Two-way ANOVA with Tukey’s multiple comparison test for behavioural data.Values of p < 0.05 were considered as significant.

## CRediT authorship contribution statement

**Francesca Picco:** Writing – original draft, Methodology, Investigation, Data curation, Conceptualization. **Lynda Zeboudj:** Methodology, Investigation, Data curation. **Silvia Oggero:** Methodology, Investigation, Data curation. **Vincenzo Prato:** Methodology, Investigation, Data curation. **Thomas Burgoyne:** Investigation. **Nikita Gamper:** Data curation. **Marzia Malcangio:** Writing – original draft, Conceptualization.

## Data availability statement

This paper does not report original code.

The raw data and any additional information required to reanalyse the data reported in this paper is available from the lead contact upon request.

## Declaration of competing interest

The authors declare that they have no known competing financial interests or personal relationships that could have appeared to influence the work reported in this paper.
